# Prolactin protects hippocampal neurons against H_2_O_2_-induced neurotoxicity by suppressing BAX and NOX4 via the NF-κB signaling pathway

**DOI:** 10.1371/journal.pone.0313328

**Published:** 2024-11-05

**Authors:** Fernando Macías, Miriam Ulloa, Carmen Clapp, Gonzalo Martínez de la Escalera, Edith Arnold

**Affiliations:** 1 Instituto de Neurobiología, Universidad Nacional Autónoma de México (UNAM), Campus UNAM-Juriquilla, Querétaro, Querétaro, México; 2 CONAHCYT–Instituto de Neurobiología, Universidad Nacional Autónoma de México (UNAM), Campus UNAM-Juriquilla, Querétaro, Querétaro, México; Georgia State University, UNITED STATES OF AMERICA

## Abstract

Reactive oxygen species (ROS) are physiological byproducts of neuronal metabolism. However, an imbalance between ROS generation and antioxidant capacity, often driven by dysregulated pro-oxidant enzymes like nicotinamide adenine dinucleotide phosphate oxidases (NOX), can result in deleterious oxidative stress. This oxidative stress is a critical factor in the pathogenesis of neurodegenerative diseases. While interventions with broad-spectrum antioxidants have demonstrated limited efficacy, the modulation of endogenous antioxidant mechanisms presents a promising therapeutic avenue. Here, we investigated the potential of the neuroprotective hormone prolactin to mitigate oxidative stress and subsequent neuronal cell death. Prolactin protected primary mouse hippocampal neurons from hydrogen peroxide (H_2_O_2_)-induced oxidative damage. Prolactin reduced ROS levels, lipid peroxidation, and apoptosis, and its effects were occluded by a specific prolactin receptor antagonist (G129R-hPRL). Mechanistically, prolactin suppressed H_2_O_2_-induced mRNA upregulation of pro-oxidative *Nox4* and pro-apoptotic *Bax*. Moreover, prolactin induced nuclear factor kappa B (NF-κB) nuclear translocation, and the inhibition of the NF-κB signaling pathway abolished the neuroprotective and transcriptional effects of prolactin, indicating its central role in prolactin-mediated protection. Our findings indicate that prolactin exerts potent antioxidant and neuroprotective effects by modulating the expression of *Nox4* and *Bax*, thereby reducing ROS generation and neuronal apoptosis. This study underscores the therapeutic potential of prolactin in attenuating oxidative stress and suggests a possible role in the treatment of neurodegenerative diseases.

## Introduction

Neurodegenerative diseases are characterized by progressive neuronal loss, often associated with increased oxidative stress-induced cell death. The presence of markers of oxidative damage has been reported in disorders such as Alzheimer’s, Parkinson’s, and Huntington’s diseases [1–3]. Although the brain maintains a physiological balance between reactive oxygen species (ROS) generation and antioxidant defenses, this equilibrium can become easily disrupted in neurons by overactivation of ROS-producing enzymes such as nicotinamide adenine dinucleotide phosphate oxidases (NOX) [4]. From the seven members of the NOX family, NOX2 and NOX4 are expressed in the hippocampus [5] and represent the main source of excess ROS that causes neuronal death and cognitive impairment in hippocampal-related neurodegenerative disorders [6–8]. Strategies based on broad-spectrum antioxidants have shown limited effectiveness [9, 10], while NOX inhibitors have low specificity, selectivity, and high toxicity to be used in humans [11]. Thus, the pursuit of alternative approaches based on the modulation of endogenous antioxidant mechanisms is crucial.

Prolactin (PRL) is a peptide hormone mainly synthetized in the anterior pituitary with established neuroprotective properties in the central nervous system (CNS) [12–17]. Its receptor is expressed in several brain regions, including the hippocampus, basal ganglia, septum, amygdala, thalamus, hypothalamus, and choroid plexus [18, 19]. In the hippocampus, PRL is capable of reducing neuronal cell death induced by kainic acid excitotoxicity [17, 20]. The mechanisms behind PRL’s effect on hippocampal neurons against excitotoxicity-induced apoptosis include the reduction of calcium overload, prevention of mitochondrial dysfunction, reduction of caspase 3-activation, and increased expression of BCL2 [21]. PRL also activates the nuclear factor kappa B (NF-κB) signaling pathway, which is implicated in the neuronal cell response to block apoptosis and can be triggered by excess ROS [21–23]. Moreover, PRL has recently attracted interest due to its potential role in mitigating oxidative stress. PRL prevents hydrogen peroxide (H_2_O_2_)-induced cell death in retinal pigmented epithelium in culture [14] and increases the expression and activity of superoxide dismutase (SOD) 1 and 2, counteracting glutamate-induced excitotoxicity in hippocampal neurons [15]. While PRL has been shown to induce the expression and activation of phagocyte NOX in fish leukocytes [24–26], its effect on NOX activation in neurons remains unclear. This study aimed to investigate the potential neuroprotective effects of PRL against oxidative damage and apoptosis in primary cultures of mouse hippocampal neurons, focusing on the involvement of NF-κB signaling and NOX expression in this process. Our findings demonstrate that PRL treatment effectively protects primary mouse hippocampal neurons against H_2_O_2_-induced oxidative damage and apoptosis. Mechanistically, PRL suppresses the upregulation of pro-oxidative *Nox4* and pro-apoptotic *Bax* via the NF-κB signaling pathway. These findings highlight the potential of PRL as a therapeutic agent for neurodegenerative diseases by modulating key pathways involved in oxidative stress-induced neuronal death.

## Materials and methods

### Animals

CD-1 pregnant dams at embryonic day 16 were anesthetized with CO_2_ and euthanized by decapitation. Next, the uterus with the live embryos was rapidly removed from the abdominal cavity, cut open, the embryos placed in ice-cold 0.1 M PBS (pH 7.4) to preserve tissue integrity and immediately sacrificed by decapitation using sharp surgical scissors. The average litter size used for each primary neuronal culture was twelve pups per female mouse. Every effort was made to minimize animal suffering and to minimize the number of animals used in order to fulfill the experimental design for proper statistical analysis. The Bioethics Committee of the Institute of Neurobiology at UNAM approved the protocol #68 used in this study in accordance with Official Mexican Standard NOM-062-ZOO-1999.

### Culture of primary hippocampal cells

Primary hippocampal neuron cultures were established as previously described [27]. Briefly, the brains of twelve embryos were obtained and hippocampi were dissected from the cortex under surgical stereomicroscopy. Hippocampi were pooled in cold dissection medium (Hank’s Balanced Salt Solution, HBSS; cat. no. 14175–095, Gibco, NY, USA) supplemented with 1 mM sodium pyruvate (cat. no. 11360070, Gibco, NY, USA), 0.1% glucose (cat. no. G-6152, Sigma-Aldrich, MO, USA), and 10 mM HEPES (pH 7.3, cat. no. H-4034, Sigma-Aldrich, MO, USA). Following dissection, hippocampi were incubated in dissection buffer containing 0.25% trypsin (cat. no. 27250–018, Gibco, NY, USA) for 20 min at 37°C. This was followed by a 10-min incubation with 1% DNase (cat. no. LS002140, Worthington-Biochem, NJ, USA) solution at room temperature. Finally, tissues were washed and disaggregated with a fire-polished Pasteur pipette, and cells were resuspended in plating medium (BME medium; cat. no. 21010–046, Gibco, NY, USA) supplemented with 10% fetal bovine serum (cat. no. 26140–079, Gibco, NY, USA), 0.45% glucose, 1 mM sodium pyruvate, 2 mM glutamine (cat. no. 35050–061, Gibco, NY, USA), and 1% penicillin/streptomycin (cat. no. L0022-100, BioWest, Nuaillé, France). Cells were seeded in 0.01% poly-L-lysine (cat. no. P4707, Sigma-Aldrich, MO, USA) coated plates or glass coverslips at a cellular density of 125,000 cells/cm^2^. Four hours later, plating medium was completely replaced by maintenance medium (Neurobasal medium; cat. no. 21103049, Gibco, NY, USA) supplemented with B-27 (1x, cat. no. 17504044, Gibco, NY, USA), 2 mM glutamine, and 1% antibiotics. One day after seeding, and in every medium change, the cultures were treated with 2 μM cytosine arabinoside (AraC; C1768-1G, Sigma-Aldrich, MO, USA). Subsequently, 50% of the medium supplemented with AraC was changed every four days. Neuronal cultures were maintained for up to 11 days *in vitro* (DIV11).

### Experimental design and cell treatments

On day *in vitro* 9 (DIV9), neuronal cultures were treated with ovine PRL (cat. no. L6520, Sigma-Aldrich, MO, USA) for 24 h. This was followed by a 24-h incubation with hydrogen peroxide (H_2_O_2_; cat. no. H1009, Sigma-Aldrich, MO, USA). Neuronal cultures plated onto glass coverslips were used for immunocytochemical studies of apoptosis; cultures plated onto 96-well plates were used for cell viability analysis and ROS quantification; cultures plated onto 6-well plates were used to investigate the effects of PRL on gene expression; and cultures plated onto 100 mm tissue culture dishes were used for lipid peroxidation quantification. The specificity of PRL actions was verified by co-incubation with a competitive PRL receptor (PRLR) antagonist (Δ1-9-G129R-hPRL; cat. no. MBS400130, MyBioSource, CA, USA) at a final concentration of 1 μM. To elucidate the participation of NF-κB, cultures were treated with the NF-κB inhibitor BAY 11–7082 (3 μM; cat. no. SML1603, Sigma-Aldrich, MO, USA) 1 h before H_2_O_2_ treatment. The number of biological replicates required in this exploratory study was estimated to three (minimum) primary hippocampal neuronal cultures.

### MTT assay

The viability of primary hippocampal neurons was assayed by measuring the reduction of tetrazolium salt, 3-(4,5-dimethylthiazol-2-yl)-2,5-diphenyltetrazolium bromide (MTT; cat. no. M2128, Sigma-Aldrich, MO, USA) to formazan crystals. MTT solution (final concentration of 5 mg/mL in PBS) was added to the neuronal culture and incubated at 37°C for 3 h. The media was aspirated, and 10% SDS in 0.01 M HCl was added to dissolve formazan crystals overnight. The absorbance of colored solutions was read by a spectrophotometer with an excitation wavelength of 570 nm (Varioskan Flash, Thermo Fisher Scientific, Inc.).

### Total reactive oxygen species detection

Intracellular ROS generation in primary cultured neurons was measured using the redox-sensitive fluorescent dye 2,7-dichlorofluorescein diacetate (DCF-DA, cat. no. D6883, Sigma-Aldrich, MO, USA) as previously described [28]. Briefly, 41.04 μM DCF-DA was added to the wells filled with 100 μl of maintenance medium and incubated for 60 minutes at 37°C. DCF-DA interacts with ROS to generate the de-acetylated fluorescent product 2,7-dichlorofluorescein (DCF). Fluorescence was read at Ex = 485 nm, Em = 530 nm (Varioskan Flash, Thermo Fisher Scientific, Inc.).

### Lipid peroxidation assay

Lipid peroxidation was assessed by determining the level of thiobarbituric acid reactive substances (TBARS) in cell lysates, as previously described [29]. Briefly, hippocampal neurons were scraped off in ice-cold PBS, lysed by sonication and centrifuged at 800 g for 5 min at 4°C. Supernatant protein was estimated using the Bradford method (cat. no. 5000006, Biorad, CA, USA). Briefly, 100 μl of supernatant and 100 μl of TRIS-HCl (20 nM, pH 7.4) were incubated at 37°C for 2 h. After incubation, 200 μl of 10% trichloroacetic acid (TCA; cat. no. T6399, Sigma-Aldrich, MO, USA) was added and centrifuged at 1000 g for 5 min at 4°C. Next, 400 μl of supernatant was transferred to a glass tube containing 400 μl of 0.5% (w/v) thiobarbituric acid (TBA; cat. no. 190284, MP Biomedicals LCC, CA, USA), and kept in boiling water for 10 min. After cooling at room temperature, the absorbance of the supernatant was determined at 532 nM (Varioskan Flash, Thermo Fisher Scientific, Inc.). TBARS were quantified using an extinction coefficient of 1.56 x 10^5^ M^-1^ cm^-1^ and expressed as nanomoles of malondialdehyde (MDA) per milligram of protein.

### TUNEL assay

Cells were fixed in 4% paraformaldehyde (cat. no. P6148, Sigma-Aldrich, MO, USA) and 4% sucrose (cat. no. 50389, Sigma-Aldrich, MO, USA) to assess DNA fragmentation using an *in situ* apoptosis detection kit (In Situ Cell Death Detection kit, fluorescein. cat. no. 11684795910, Roche Diagnostics, Manheim, Germany) according to the manufacturer’s instructions. Terminal deoxynucleotidyl transferase incorporated nucleotide was labeled with fluorescein, and DAPI-supplemented mounting medium was used for nuclear counterstaining. Digital images were captured with a Zeiss LSM 780 confocal microscope (Carl Zeiss, Germany), and TUNEL-positive cells were counted with Image J software.

### Immunocytochemistry and image acquisition

The general immunocytochemistry method was followed as previously described [27]. Hippocampal neurons previously fixed in 4% paraformaldehyde and 4% sucrose at DIV11 were permeabilized using 0.1% Triton X-100 (cat. no. X198-07, J.T. Baker, NJ, USA), followed by blocking with 10% normal goat serum (cat. no. 10000C, Gibco, NY, USA) for 1 h at room temperature. Incubation with primary monoclonal antibody anti-p65 (1:300; cat. no. sc-8008, RRID: AB_628017, Santa Cruz Biotechnology, CA, USA) was carried out overnight at 4°C. Subsequently, coverslips were incubated with secondary antibody with Texas Red goat anti-mouse (dilution 1:1000, cat. no. T-862, Molecular Probes, Invitrogen, CA, USA) for 1 h at room temperature. Nuclei were counterstained with SYTOX^TM^ Green (cat. no. S7020, Thermo Fisher Scientific, MA, USA). Digital images were acquired using a Zeiss LSM 780 confocal microscope (Carl Zeiss, Germany). Z-stack images were captured from a single position and then compiled into a single 2D image using maximum intensity projection. Fluorescence quantification was conducted using ImageJ 1.51 (NIH) software.

### RNA isolation and cDNA synthesis

Total RNA was extracted using RNeasy Mini Kit (cat. no. 74106; Qiagen, CA, USA) from primary cultured hippocampal neurons according to the manufacturer’s protocol. For each sample, 1 μg of total RNA was subjected to reverse transcription using the High-Capacity cDNA Reverse Transcription Kit (cat. no. 10400745, Applied Biosystems, CA, USA) according to the manufacturer’s instructions.

### Quantitative PCR

Polymerase chain reaction (PCR) products were detected and quantified with Maxima SYBR Green/ROX qPCR Master Mix (cat. no. K0223, Thermo Fisher Scientific, AL, USA) in a 10-μl final reaction volume containing template and 0.5 μM of each primer (Table 1). Amplification was performed in the CFX96 real-time PCR detection system (Bio-Rad, Hercules, CA). Conditions were 15 s at 95°C, 30 s at the primer pair-annealing temperature, and 30 s at 72°C for 40 cycles. Relative mRNA levels were calculated using the comparative 2^-ΔΔCt^ method, and cycle thresholds were normalized to the housekeeping gene hypoxanthine-guanine phosphoribosyltransferase (*Hprt*).

**Table 1:** Primers used for real-time PCR.

	NCBI accession number	Direction	Sequence	Amplicon size (bp)
*Bax*	NM_007527.3	F	AAGAAGCTGAGCGAGTGTCT	179
		R	AAGTAGAAGAGGGCAACCAC	
*Bcl2*	NM_009741.5	F	CTGTGGATGACTGAGTACCT	184
		R	GGTATGCACCCAGAGTGAT	
*Hprt*	NM_013556.2	F	TTGCTGACCTGCTGGATTAC	148
		R	GTTGAGAGATCATCTCCACC	
*Nox2*	NM_007807.5	F	TCCTATGTTCCTGTACCTTTGTG	122
		R	GTCCCACCTCCATCTTGAATC	
*Nox4*	NM_015760.5	F	CTCTACTGGATGACTGGAAACC	141
		R	AGTCAGGTCTGTTTTCTTGCC	
*Prlr*	NM_011169.5	F	GGAAACATTCACCTGCTGGT	163
		R	TATGGAAGTGTACTGCTTGCT	
*Rbfox3*	NM_001039168.1	F	GGGCCGTGCTGTGTATAAT	139
		R	TGGCTGAGCATATCTGTAAGC	

### Statistical analysis

Statistical analysis was performed using GraphPad Prism 8.0.1 software (GraphPad Software, La Jolla, CA). Data were assessed for normality and homogeneity of variance using the Shapiro-Wilk test. One-way analysis of variance (ANOVA) with Tukey’s HSD post-hoc test was used for normally distributed data with equal variances. The Kruskal-Wallis and Mann-Whitney U tests were used for non-normally distributed data or unequal variances. Student’s t-test (two-tailed) compared differences between two groups. Data are means ± SEM. The value of "n" represents the number of independent cultures. For imaging data, multiple images per culture were acquired and averaged before analysis. Differences were considered statistically significant at *p* < 0.05.

## Results

### PRL protects mouse hippocampal neurons against H_2_O_2_-induced cell death and lipid peroxidation

**Fig 1:**
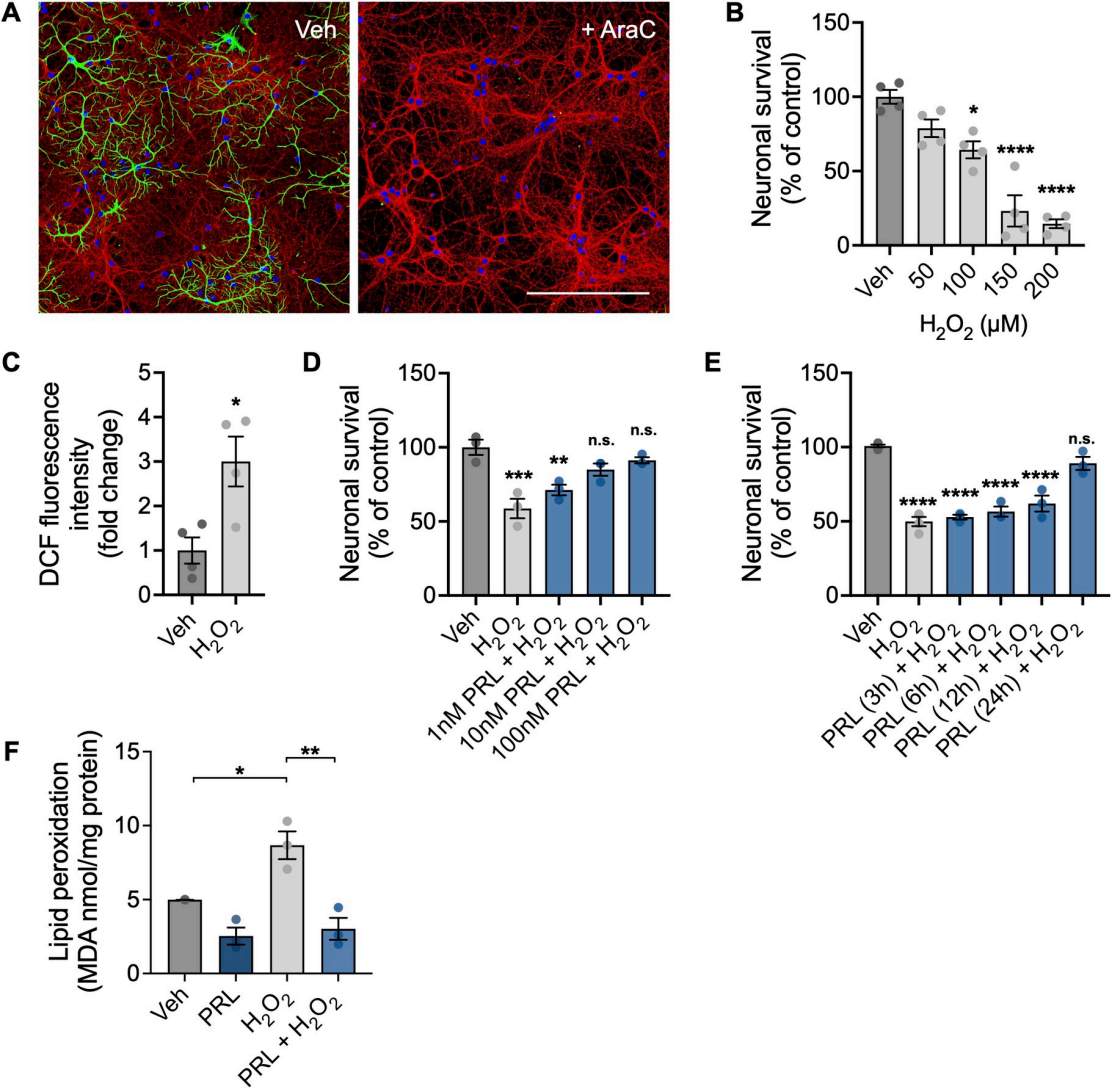
Effect of PRL on H_2_O_2_-induced cell death and oxidative damage in mouse hippocampal neurons. (A) Representative images taken on day *in vitro* 11 from mouse hippocampal neuron cultures treated or not with 2 μM Cytosine β-D-arabinofuranoside (AraC) starting on day *in vitro* 1. Scale bar 200 μm. (B) Hippocampal neurons were incubated for 24 h with increasing concentrations (50–200 μM) of hydrogen peroxide (H_2_O_2_). Cell viability was assessed by the MTT assay and normalized to the vehicle control. (n = 4). (C) Hippocampal neurons were incubated with 100 μM H_2_O_2_ for 60 min, and generation of reactive oxygen species (ROS) was quantified using 2’,7’-dichlorodihydrofluorescein diacetate (DCF-DA). ROS levels are expressed as DCF fluorescence after 30 min of incubation with DCF-DA. (n = 4). (D) Hippocampal neurons were pre-incubated with increasing concentrations (1–100 nM) of prolactin (PRL) or vehicle for 24 h, followed by treatment with 100 μM H_2_O_2_ or vehicle for 24 h. Cell viability was quantified by the MTT assay, and the results were normalized to the vehicle control. (n = 3). (E) Hippocampal neurons were pre-incubated for 24 h with 100 nM PRL or vehicle, followed by treatment with 100 μM H_2_O_2_ or vehicle for 24 h. Lipid peroxidation was determined by measuring malondialdehyde (MDA), a thiobarbituric acid reactive substance (TBARS). MDA concentration was normalized to total protein content. *p<0.05, **p<0.01, ***p<0.001, ****p<0.0001 vs vehicle or indicated group; n.s., non-significant.

In order to establish highly pure primary hippocampal neuronal cultures, cells were treated from DIV1 to DIV11 with the anti-mitotic drug AraC (2 μM) to eliminate the glial cell population. AraC treatment successfully reduced glial fibrillary acidic protein-positive cells in hippocampal neuronal cultures (Fig 1A). Treatment with H_2_O_2_ at DIV10 induced a dose-dependent reduction in neuronal survival as measured by the MTT assay 24 h later. A 100 μM H_2_O_2_ dose was found to reduce cell viability from 100 ± 9.375% in control conditions to 64.32 ± 11.36% (F_(4,15)_ = 0.8795; *p = 0*.*0106*; Fig 1B) and significantly induced a three-fold increase in ROS generation analyzed 60 min after treatment (*p =* 0.0195; Fig 1C). To assess the protective effects of PRL, 100 μM H_2_O_2_ was used in subsequent experiments to induce significant oxidative stress while preserving approximately half of the neuron culture viability. Pretreatment for 24 h with PRL significantly inhibited H_2_O_2_-induced cell death at a concentration of 10 nM (84.93 ± 7.199 vs 58.78 ± 11.36%; F _(4,10)_ = 12.88; *p = 0*.*0152*; Fig 1D) and 100 nM PRL (91.23 ± 3.562 vs 58.78 ± 11.36%; *p = 0*.*0036*; Fig 1D). On the basis of these results, a PRL concentration of 100 nM was selected to evaluate the time course of its neuroprotective effect. Neurons were pretreated with PRL for 3, 6, 12, and 24 h. Only the 24-h PRL treatment significantly inhibited H_2_O_2_-induced cell death (89.03 ± 7.534 vs 49.89 ± 6.252%; F _(5,14)_ = 45.14; *p < 0*.*0001;* Fig 1E). Lipid peroxidation was determined by measuring TBARS content expressed as the level of MDA. A significant 1.7-fold increase in MDA content was observed in neurons treated with 100 μM H_2_O_2_ in comparison with vehicle-treated cultures (8.672 ± 1.621 vs 4.988 ± 0.01 nmol/mg protein; F _(3,8)_ = 17.65; *p = 0*.*0183*; Fig 1F). PRL pretreatment prevented an increase in MDA in H_2_O_2_-treated neurons (3.035 ± 1.288 vs 8.672 ± 1.621 nmol/mg protein; *p = 0*.*0014*; Fig 1F).

### PRL prevents H_2_O_2_-induced cell death and ROS generation through the activation of its receptor in mouse hippocampal neurons

**Fig 2:**
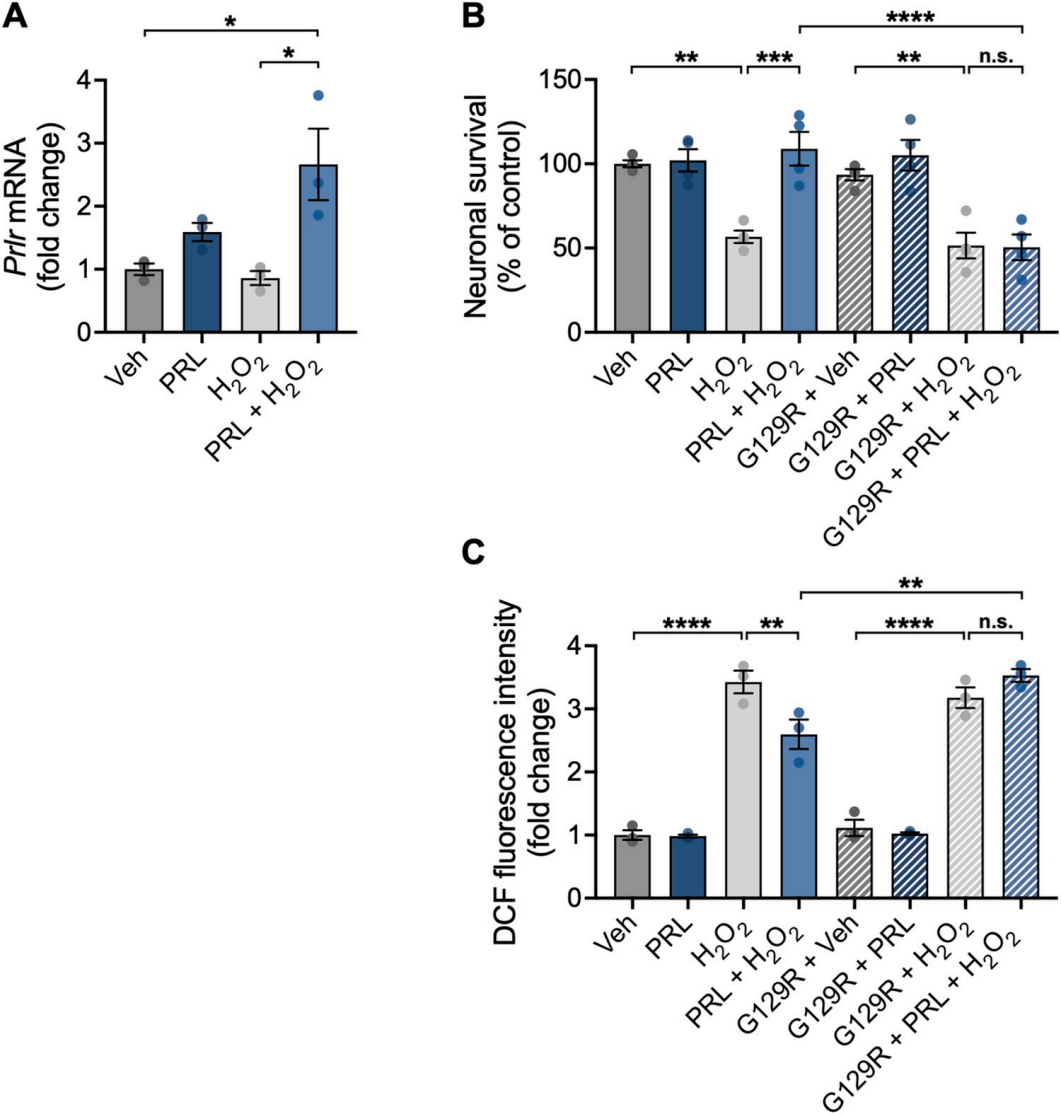
Effect of the functional antagonism of the PRLR in the protective effect of PRL on mouse hippocampal neurons. (A) Hippocampal neurons were pre-incubated for 24 h with 100 nM prolactin (PRL) or vehicle, followed by treatment with 100 μM H_2_O_2_ or vehicle for 24 h. The prolactin receptor (*Prlr*) mRNA level was measured by quantitative RT-PCR. Data were initially normalized using the *Hprt* housekeeping gene as an internal control and then nornalized to the corresponding gene expression in the vehicle-treated group. Hippocampal neurons were pre-incubated for 24 h with 100 nM PRL or vehicle in the presence or absence of 1 μM of the competitive PRLR antagonist 1–9 G129R hPRL (G129R). Subsequently, cells were exposed to 100 μM H_2_O_2_ or vehicle for an additional 24 h. (B) Cell viability was assessed by the MTT assay and normalized to the vehicle control. (C) Generation of reactive oxygen species (ROS) was quantified using 2’,7’-dichlorodihydrofluorescein diacetate (DCF-DA). ROS levels are expressed as DCF fluorescence after 30 min of incubation with DCF-DA. (A-C). (n = 3). *p<0.05, **p<0.01, ***p<0.001, ****p<0.0001 vs vehicle or indicated group; n.s., non-significant.

Treatment with PRL increased *Prlr* mRNA expression in neuronal cultures treated with H_2_O_2_ (2.663 ± 0.9834 vs 1.0 ± 0.1587 fold change; F _(3,8)_ = 7.393; *p = 0*.*0190*; Fig 2A) in comparison to control conditions, suggesting that PRL may upregulate its own receptor to amplify its effects on neurons under a detrimental insult. Then, we corroborated that the observed protective actions of PRL were mediated via its receptor. Neurons were treated with PRL in the presence or absence of the PRLR antagonist 1–9 G129R hPRL at a concentration 10-fold higher than PRL (1 μM), which has been reported to block PRLR signaling [30]. Co-treatment with 1–9 G129R hPRL completely blocked PRL’s effect on both neuronal survival (50.44 ± 15.29 vs 108.0 ± 20.03%; F _(7, 24)_ = 14.35; *p < 0*.*001;* Fig 2B) and ROS generation (3.53 ± 0.1769 vs 2.597 ± 0.40 AU; F _(7, 16)_ = 75.75; *p = 0*.*0033;* Fig 2B) in cultures treated with H_2_O_2_.

### NF-κB activation is involved in PRL-mediated protection from H_2_O_2_-induced cell death

**Fig 3:**
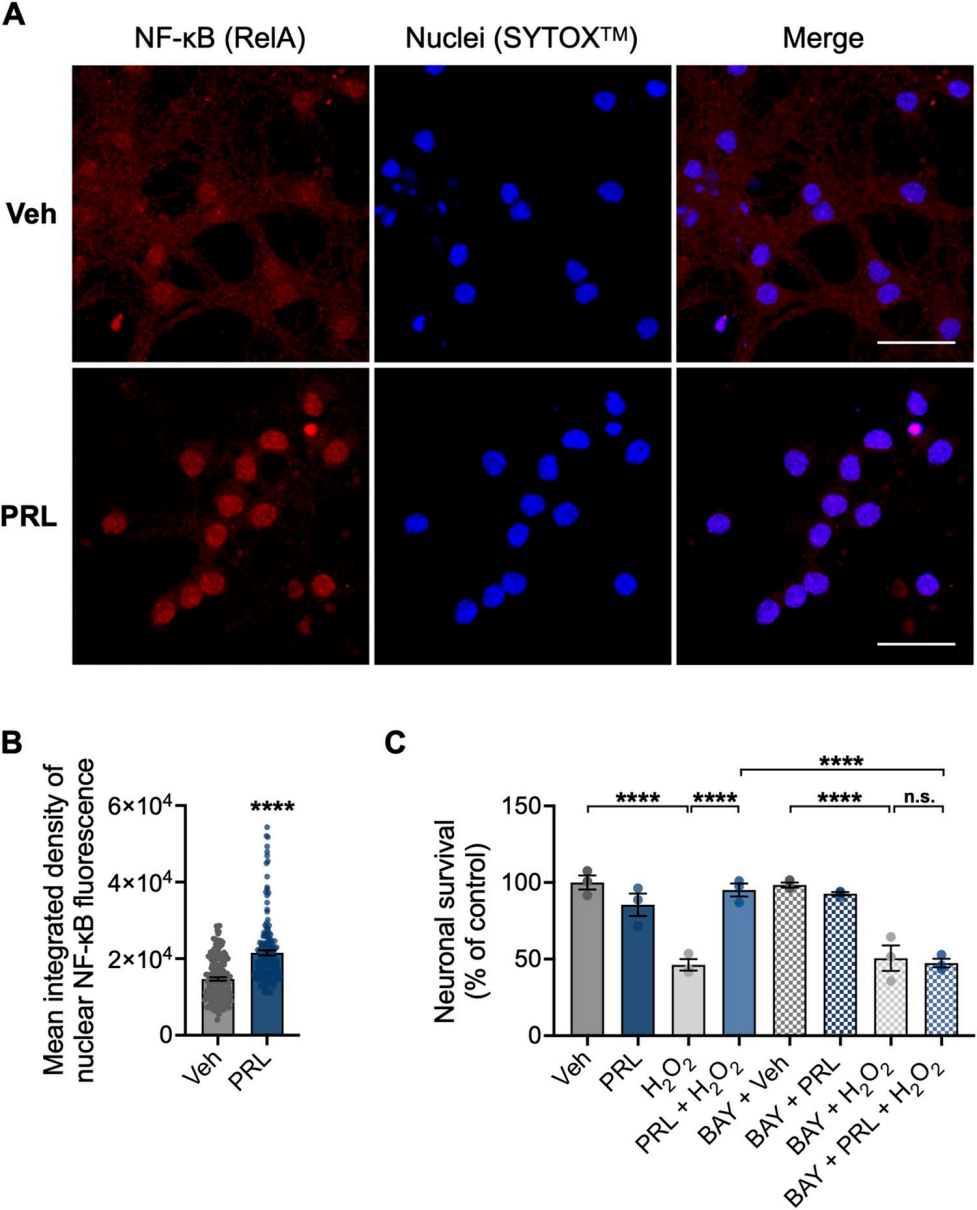
Involvement of NF-κB in the protective effect of PRL on mouse hippocampal neurons. (A) Representative nuclear factor kappa B (NF-κB) immunostaining images of mouse hippocampal neurons treated with vehicle or 100 nM prolactin (PRL) for 48 h. Right panels are merged images of NF-κB (red, left panels) and the nuclear marker SYTOX^TM^ (blue, center panels). Scale bar 20 μm. (B) Quantification of nuclear NF-κB in control versus PRL-exposed hippocampal neurons. (n = 3). (C) Hippocampal neurons were pre-incubated for 24 h with 100 nM PRL or vehicle, followed or not by treatment with 3 μM of the NF-κB inhibitor BAY 11–7082 (BAY) 1 h before treatment with 100 μM H_2_O_2_ or vehicle for 24 h. Cell viability was assessed by the MTT assay and normalized to the vehicle control. (n = 3). ****p<0.0001 vs vehicle or indicated group; n.s., non-significant.

Previous studes have demonstrated that PRL can activate NF-κB in neurons [21]. Based on these findings, we investigated whether NF-κB signaling is involved in the PRL-mediated protective mechanism in mouse hippocampal neurons. PRL treatment elicited a significant accumulation of the NF-κB p65 subunit in neurons nuclei (19732 ± 5453 vs 14686 ± 5761; p < 0.001; Fig 3A and 3B), suggesting that PRL induced the nuclear translocation of NF-κB. In view of this, NF-κB involvement in the protection of PRL against H_2_O_2_-induced cell death in hippocampal neurons was pharmacologically evaluated using BAY 11–7082, which inhibits the activity of the inhibitor of nuclear factor-κB (IκB) kinase (IKK). This inhibition maintains NF-κB in its inactive complex with IkB [31]. BAY 11–7082 was used at 3 μM, a nontoxic concentration for neurons (S1 Fig). PRL protection against H_2_O_2_-induced loss of cell viability was abolished when 3 μM BAY 11–7082 was added to the cells 1 h before H_2_O_2_ treatment (47.39 ± 4.883 vs 95.16 ± 7.337%; F _(7, 16)_ = 25.30; *p < 0*.*001*; Fig 3C). The NF-κB inhibitor alone had no effect on the viability of hippocampal neurons (Fig 3C).

### PRL down-regulates H_2_O_2_-induced *Bax* gene expression through NF-κB signaling

**Fig 4:**
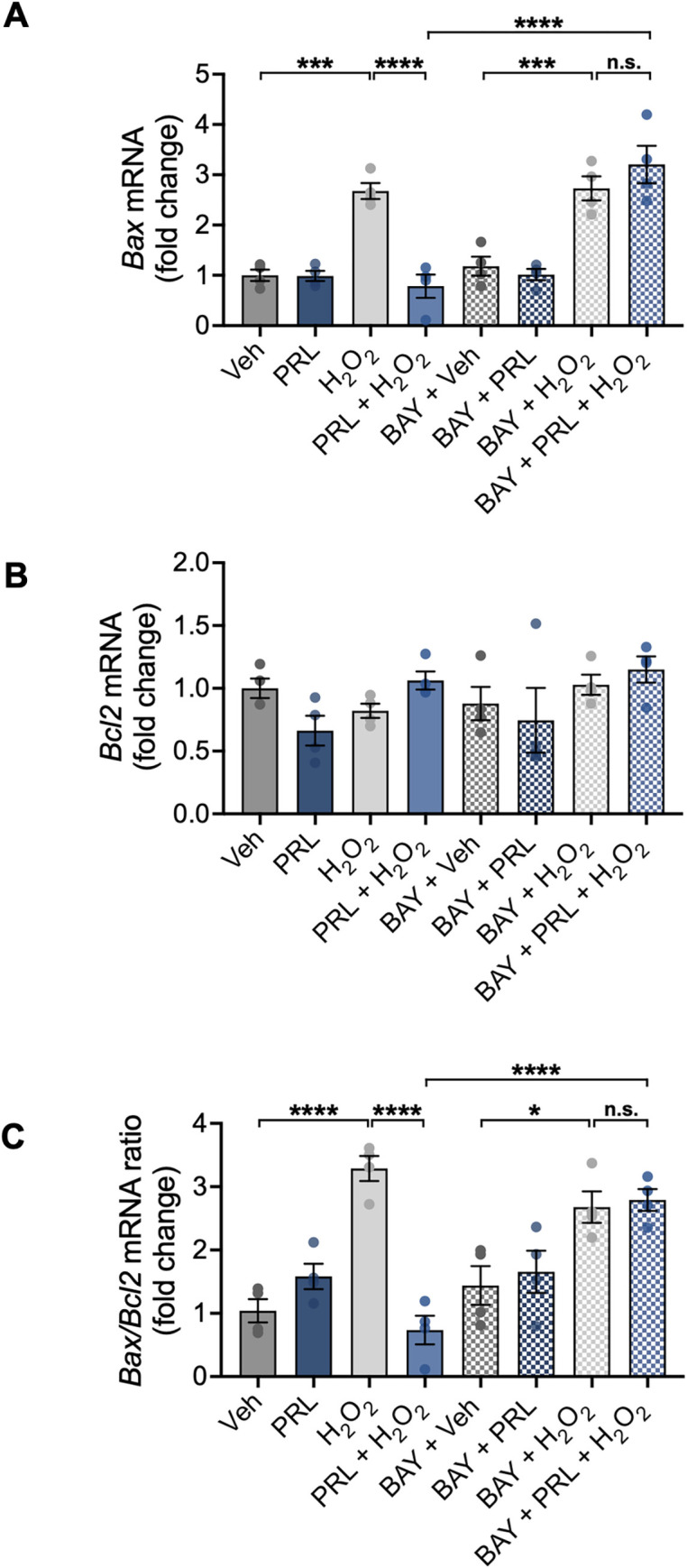
Effect of PRL on transcription of anti- and pro-apoptotic genes in mouse hippocampal neurons. Hippocampal neurons were pre-incubated for 24 h with 100 nM prolactin (PRL) or vehicle, followed or not by treatment with 3 μM of the nuclear factor kappa B (NF-κB) inhibitor BAY 11–7082 (BAY) 1 h before treatment with 100 μM H_2_O_2_ or vehicle for 24 h. mRNA levels of (A) *Bax* and (B) *Bcl2* were measured by quantitative RT-PCR. Data were initially normalized using the *Hprt* housekeeping gene as an internal control and then normalized to the corresponding gene expression in the vehicle-treated group. (C) Ratio of *Bax* and *Bcl2* expression in hippocampal neurons under the evaluated conditions. (n = 4). *p<0.05, ***p<0.001, ****p<0.0001 vs vehicle or indicated group; n.s., non-significant.

The expression of *Bax* and *Bcl2* mRNA was analyzed to determine whether the observed anti-apoptotic effect of PRL involves direct influence on the intrinsic apoptosis pathway. Treatment with H_2_O_2_ resulted in a 2.67-fold increase in *Bax* expression relative to control conditions (2.676 ± 0.3176 vs 1.0 ± 0.2235 fold change; F _(7, 24)_ = 22.72; *p < 0*.*001*; Fig 4A). PRL significantly reduced the H_2_O_2_-induced elevation in *Bax* expression (0.7843 ± 0.4607 vs 2.676 ± 0.3176, *p < 0*.*001*; Fig 4A). On the other hand, *Bcl2* expression did not change in response to H_2_O_2_, PRL, or NF-κB inhibition (Fig 4B). The *Bax/Bcl2* ratio increased after H_2_O_2_ treatment (3.286 ± 0.3954 vs 1.039 ± 0.3671 fold change; F _(7, 24)_ = 14.40; *p < 0*.*001*; Fig 4C), and PRL blocked this increase (0.7348 ± 0.4515 vs 3.286 ± 0.3954 fold change *p < 0*.*001*; Fig 4C). These results show that PRL prevents the activation of the intrinsic apoptosis pathway. BAY 11–7082 blocked the PRL-mediated reduction in *Bax* expression (2.728 ± 0.4792 vs 0.7843 ± 0.4607, *p < 0*.*001;* Fig 4C) and the *Bax/Bcl2* ratio (2.790 ± 0.3452 vs 0.7348 ± 0.4515, p < 0.001; Fig 4C). The concentration of BAY used in our study was not cytotoxic (S1 Fig), resulting in relatively unchanged *Bax* expression in the BAY+Veh group. This suggests that BAY alone does not significantly alter basal NF-κB-mediated protection. Consequently, the observed effects in the BAY+H_2_O_2_ group primarily reflect the influence of H_2_O_2_ on *Bax* expression. Furthermore, the absence of a statistically significant difference between H_2_O_2_ and BAY+PRL+H_2_O_2_ groups indicates that BAY primarily blocks the pro-survival effects induced by PRL. These findings support the role of NF-κB in PRL’s neuroprotective effects.

### PRL prevents H_2_O_2_-induced apoptosis in mouse hippocampal neurons

**Fig 5:**
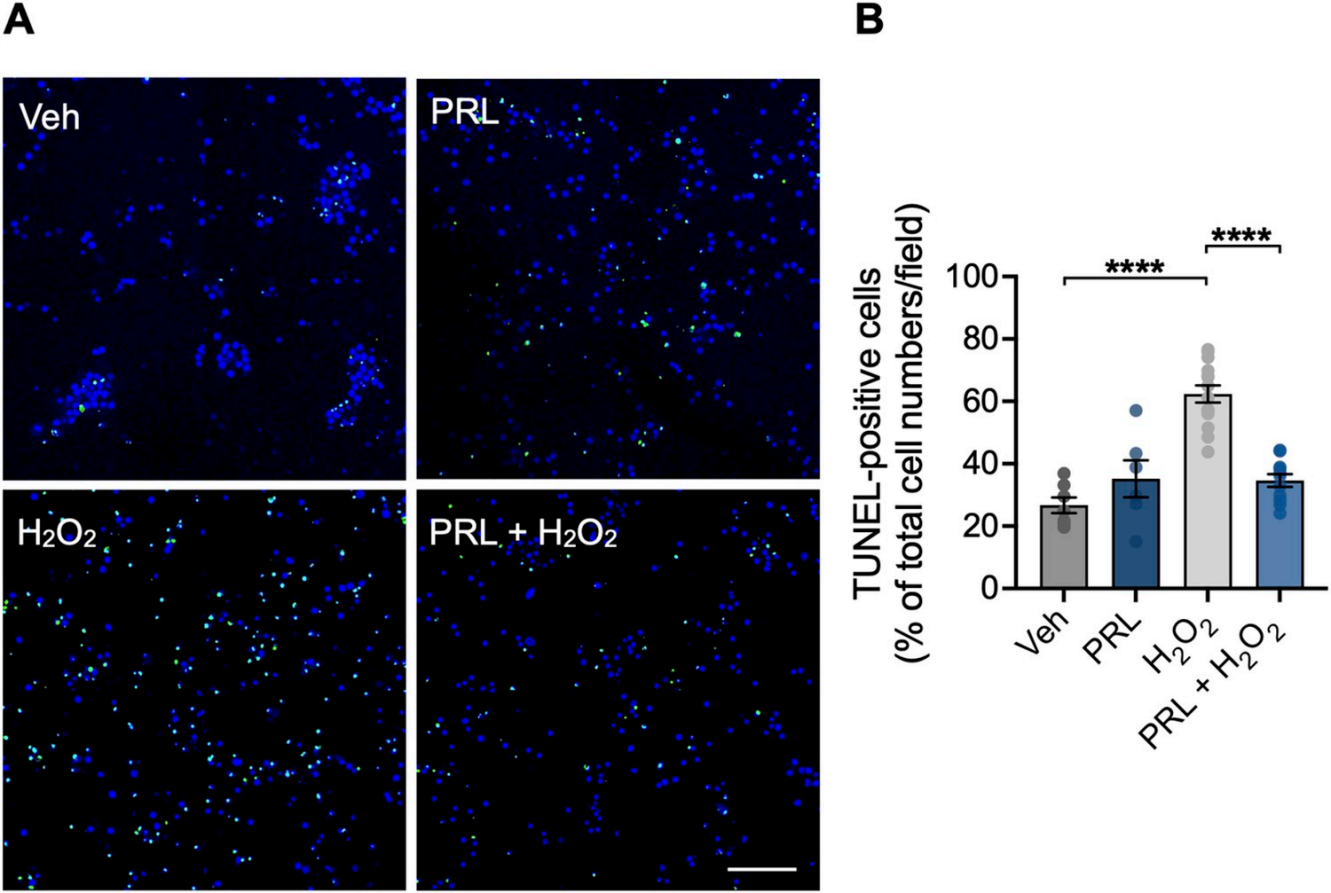
Effect of PRL on H_2_O_2_-induced apoptosis in mouse hippocampal neurons. Hippocampal neurons were pre-incubated for 24 h with 100 nM prolactin (PRL) or vehicle, followed by treatment with 100 μM H_2_O_2_ or vehicle for 24 h. Cells were stained for apoptosis using the TUNEL assay (green), and nuclei were conterstained with DAPI (blue). (A) Representative images of TUNEL staining in hippocampal neuronal cultures treated with vehicle, PRL, and H_2_O_2_. Scale bar 200 μm. (B) Quantification of TUNEL-positive cells. Bar plot shows the percentage of neurons positive to TUNEL staining per image. (n = 3). ****p<0.0001 vs vehicle or indicated group.

Previous studies have shown that a pro-oxidant insult with H_2_O_2_ promotes death by apoptosis in neurons [32]. To investigate if PRL reduces apoptosis in hippocampal neurons treated with H_2_O_2_, DNA fragmentation was tested using the TUNEL assay. Compared to vehicle-treated cultures, H_2_O_2_ treatment significantly increased the percentage of TUNEL-positive cells (62.34 ± 10.37 vs 26.69 ± 6.595%; F _(3,34)_ = 29.31; *p < 0*.*001*; Fig 5A and 5B). PRL pretreatment prevented this H_2_O_2_-induced increase in apoptotic cells (34.63 ± 6.809 vs 62.34 ± 10.37; *p < 0*.*001*; Fig 5A and 5B).

### PRL down-regulates H_2_O_2_-induced *Nox4* gene expression through NF-κB signaling

**Fig 6:**
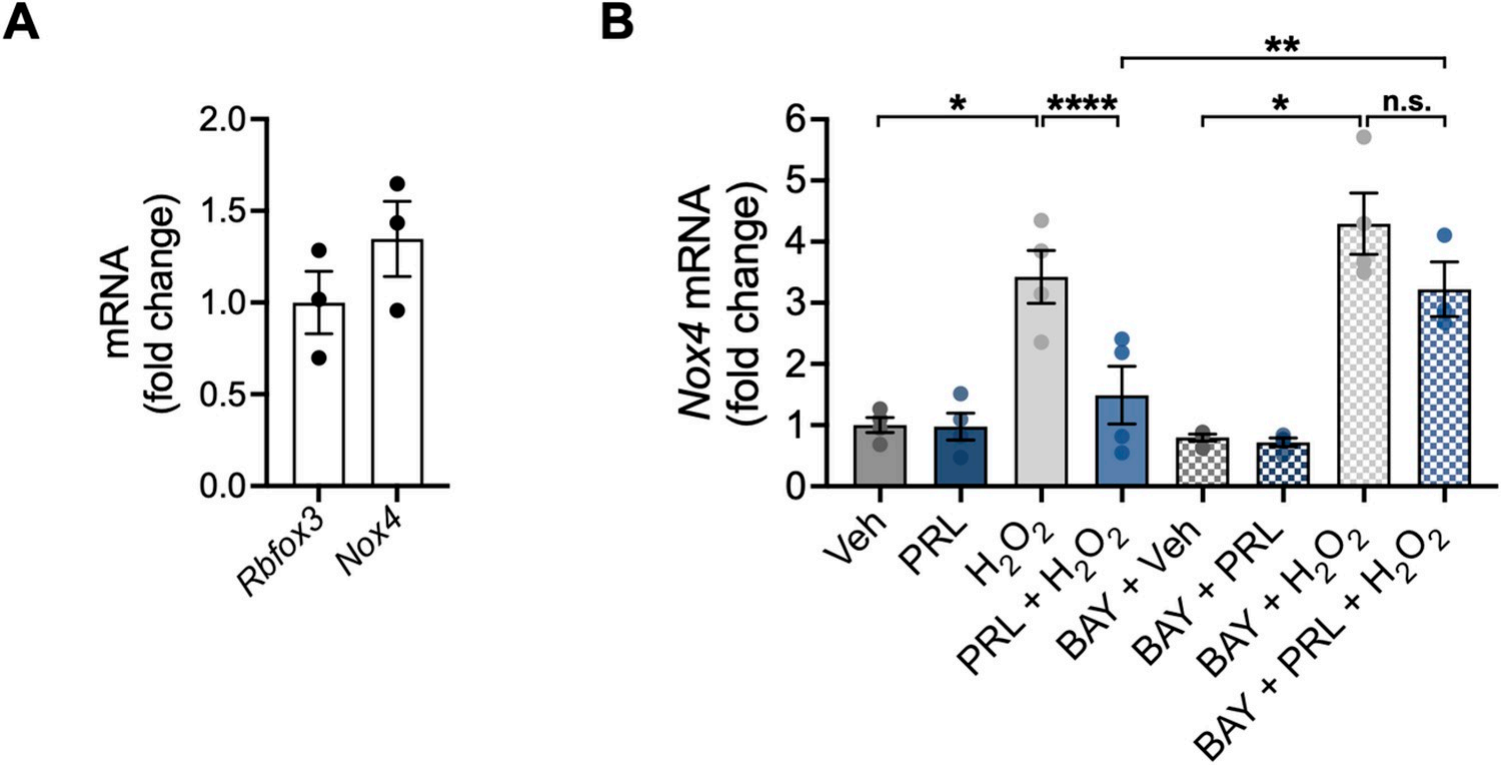
Effect of PRL on the transcription of NOX genes in mouse hippocampal neurons. Hippocampal neurons were pre-incubated for 24 h with 100 nM prolactin (PRL) or vehicle, followed or not by treatment with 3 μM of the nuclear factor kappa B (NF-κB) inhibitor BAY-117082 (BAY) 1 h before treatment with 100 μM H_2_O_2_ or vehicle for 24 h. (A) mRNA levels of *Rbfox3* and *Nox4* in hippocampal neurons treated with vehicle were measured by quantitative RT-PCR. Data were initially normalized using the *Hprt* housekeeping gene as an internal control. *Nox4* expression was further normalized to the corresponding *Rbfox3* expression. (B) *Nox4* mRNA expression was measured by quantitative RT-PCR in hippocampal neurons under all treatment conditions. (A) (n = 3) or (B) (n = 4). *p<0.05, **p<0.01, ****p<0.0001 vs vehicle or indicated group; n.s., non-significant.

Given that NOX enzymes are an endogenous source of ROS [4], we assessed changes in the mRNA expression of NOX isoforms *Nox2* and *Nox4*. Under control conditions, we found a positive correlation between *Nox4* expression and hippocampal neuronal density, as estimated by NeuN (*Rbfox3*) expression (Fig 6A). *Nox2* expression was practically undetectable (Ct > 35) when normalized to NeuN (*Rbfox3*) expression (S2 Fig). Neurons treated with 100 μM H_2_O_2_ had a significant 3.4-fold increase in *Nox4* expression (3.423 ± 0.8664 vs 1.0 ± 0.246 fold change; F _(7, 23)_ = 18.10; *p < 0*.*001*; Fig 6B), which was prevented by PRL pretreatment (1.490 ± 0.9422 vs 3.423 ± 0.8664 fold change; *p = 0*.*0079*; Fig 6B). To investigate whether NF-κB is involved in PRL-induced transcriptional changes of *Nox4* mRNA expression, NF-κB signaling was inhibited with BAY 11–7082 before H_2_O_2_ treatment. BAY 11–7082 blocked the reduction in *Nox4* mRNA expression observed in PRL-pretreated neurons in response to H_2_O_2_ treatment (3.221 ± 0.7744 vs 1.490 ± 0.9422 fold change; *p = 0*.*0394*; Fig 6B).

## Discussion

**Fig 7:**
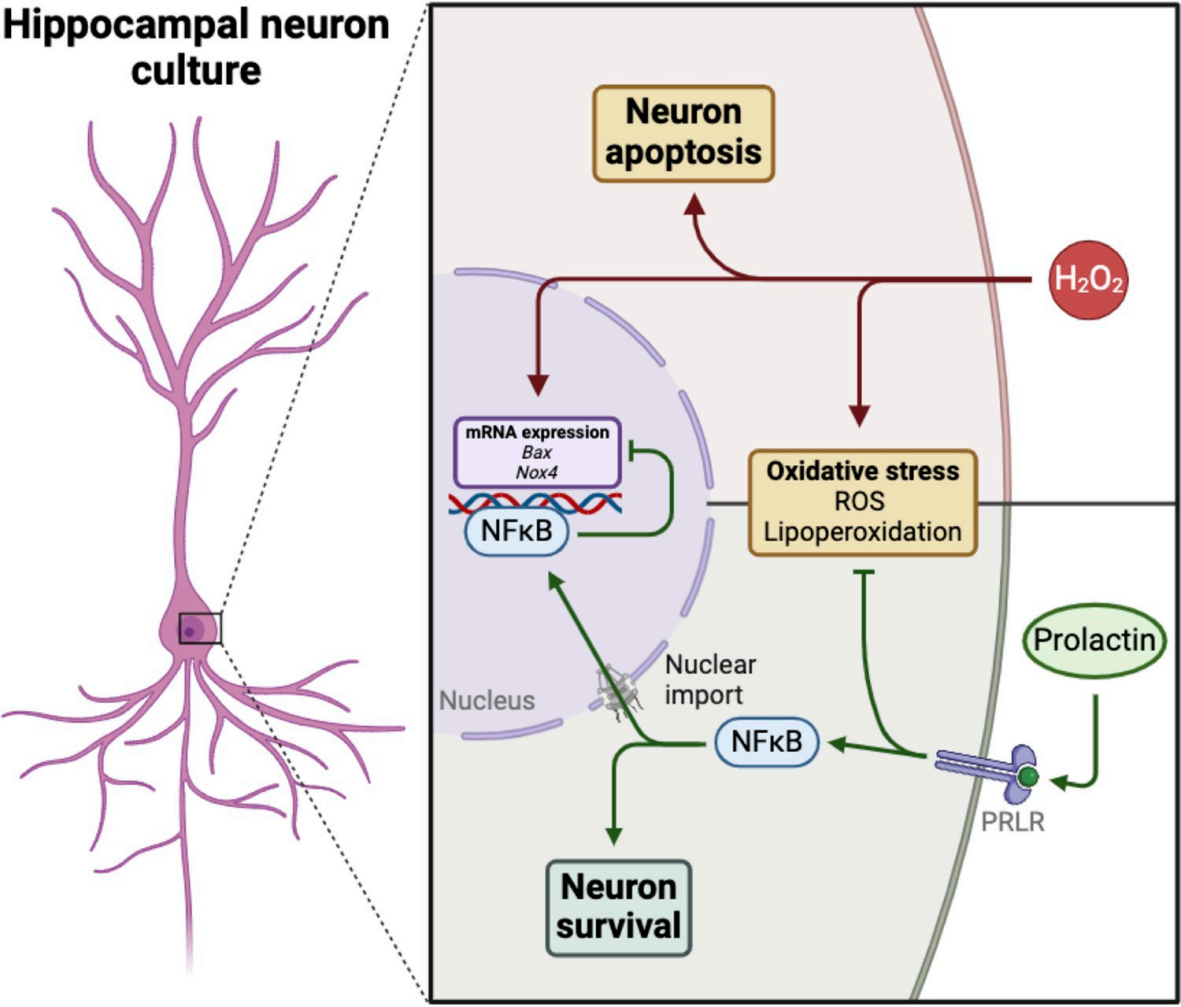
Schematic representation of PRL’s protective effects against H_2_O_2_ neurotoxicity in hippocampal neurons. PRL binding to its receptor (PRLR) triggers the nuclear import of nuclear factor kappa B (NF-κB), leading to the downregulation of pro-apoptotic *B*ax and pro-oxidant *Nox4* expression induced by hydrogen peroxide (H_2_O_2_). This mechanism could underlie PRL’s ability to promote neuron survival by reducing reactive oxygen species (ROS) generation, lipoperoxidation, and apoptosis triggered by H_2_O_2_ (Created with BioRender.com).

Exploring molecules with neuroprotective potential and identifying the pathways that prevent oxidative stress-induced cell death is essential to uncovering putative therapies for neurodegenerative diseases. PRL, a well-established neuroprotective hormone, has recently been recognized for its antioxidant properties within the CNS, as evidenced by its role in enhancing astrocyte antioxidant capacity [33] and protecting the retinal pigmented epithelium from H_2_O_2_-induced oxidative stress [14]. However, the direct antioxidant effects of PRL on neurons remain largely unexplored. To address this knowledge gap, we investigated the impact of PRL on H_2_O_2_-induced oxidative stress in hippocampal neuronal cultures. Our findings demonstrate that activation of the PRLR signaling cascade protects hippocampal neurons challenged by H_2_O_2_. PRL exerts this neuroprotective effect through activation of the NF-κB signaling pathway. This activation leads to decreased expression of the pro-oxidant enzyme NOX4 and the pro-apoptotic protein BAX, therefore preventing ROS production, reducing lipid peroxidation, inhibiting mitochondrial pore formation, and ultimately preventing neuronal cell death (Fig 7).

Prior research has shown that PRL has neuroprotective effects against excitotoxic hippocampal damage *in vivo* and can limit glutamate-induced lipid peroxidation in hippocampal neuronal cultures [15, 17, 20, 21, 34–36]. However, its direct effect on ROS-induced neuronal cell death has remained unexplored. Our study demonstrates that PRL treatment significantly prevents apoptosis in cultured hippocampal neurons exposed to H_2_O_2_. This finding highlights a novel role for PRL in protecting neurons from oxidative stress. PRL likely exerts its neuroprotective effect through an antioxidant mechanism, as PRL treatment reduces ROS levels, and this effect is blocked by a competitive antagonist of its receptor. Moreover, PRL suppresses lipid peroxidation, a major contributor to neuronal cell death during oxidative stress. Broad spectrum antioxidants (e.g., vitamin E) and hormones that suppress lipid peroxidation, such as sex steroids, are known to protect neurons *in vitro* [37, 38]. Notably, our findings align with previous reports demonstrating that PRL treatment protects both retinal pigmented epithelium and astrocytes cultures from H_2_O_2_-induced cell death by reducing ROS levels [14]. These findings strengthen the generalizability of PRL’s antioxidant potential across different CNS cell types.

Our findings suggest that a longer incubation period (24 h) is required to fully achieve PRL’s protective effects. This implies that the underlying mechanisms of PRL’s neuroprotection may involve transcriptional events. To elucidate the molecular mechanism by which PRL prevents H_2_O_2_-induced cell death, we assessed the role of NF-κB, a redox-sensitive transcription factor known to regulate genes involved in neuronal survival [39]. Our data suggest that NF-κB mediates the anti-apoptotic actions of PRL in hippocampal neuron cultures exposed to H_2_O_2_. Consistent with previous work [15], PRL treatment induced NF-κB nuclear translocation in hippocampal neuronal cultures, indicating activation. Furthermore, the neuroprotective effect of PRL was abolished in cultures cotreated with NF-κB inhibitor (BAY 11–7082). These findings align with previous studies demonstrating that pretreatment with NF-κB activators (e.g., TNFα and C2 ceramide) protects hippocampal neuron cultures from oxidative stress-induced apoptosis [23]. This suggests that PRL’s neuroprotection might include the NF-κB-mediated regulation of genes involved in cell survival. We investigated the expression of the anti-apoptotic protein BCL2 and the pro-apoptotic protein BAX, both of which are regulated by NF-κB in hypoxia- and glutamate-induced *in vitro* models of neuronal apoptosis [40–42]. Interestingly, neither PRL treatment nor NF-κB inhibition affected *Bcl2* mRNA expression in hippocampal neuron cultures. However, PRL did block the H_2_O_2_-induced increase in *Bax* in hippocampal neuron cultures, and this effect depended on NF-κB activation. BAX can dimerize with itself or with BCL2, and when overproduced, BAX homodimers promote the formation of pores in the mitochondrial membrane, leading to cell death [43]. By blocking *Bax* upregulation, PRL decreased the ratio of *Bax* to *Bcl2* expression, thereby increasing the cellular resistance to apoptotic stimuli and reducing cell death. While the downregulation of *Bax* provides a clear target for PRL’s neuroprotective effect, investigating potential antioxidant signaling pathways is crucial for a more comprehensive understanding of the mechanism.

H_2_O_2_-induced oxidative stress is mediated, in part, by the activation of the pro-oxidant NOX enzymes. Among the seven NOX isoforms, NOX2 and NOX4 play an important role in neuronal ROS production under injury conditions [44, 45]. NOX4 is particularly relevant, as it constitutively produces H_2_O_2_ and its activity is mainly regulated at the mRNA level [46, 47]. Furthermore, NOX4 can exhibit a dual role depending on the neuronal cell context. Under physiological basal conditions, NOX4 levels contribute to neuroprotection by regulating ROS and calcium homeostasis, thus preventing neuronal hyperexcitability and subsequent cell death *in vivo* [48]. In contrast, under pathological conditions associated with ROS excess, NOX4 expression is upregulated and contributes to neuronal cell death [49, 50]. Accordingly, knocking down NOX4 in neurons effectively reduced neurotoxicity and prevented cognitive decline in a taupathy mouse model [51]. Our findings support this notion, since *Nox4* expression under basal conditions appears to be proportionate to hippocampal neuronal density as revealed by *NeuN* expression, suggesting a physiological requeriment. Conversely, H_2_O_2_ treatment significantly increased *Nox4* mRNA expression in hippocampal neurons, an effect that was abolished by PRL. Notably, this effect of PRL was dependent on NF-κB activation, as co-treatment with the NF-κB inhibitor (BAY 11–7082) blocked the decrease in *Nox4* mRNA expression. Although NOX4 has been repeatedly implicated in the progression of Alzheimer’s disease and Parkinson’s disease [52, 53], its upregulation occurs primaily in astrocytes. This suggests a broader role for NOX4 in neurodegenerative processes, both in astrocytes and neurons, reinforcing its potential as a therapeutic target. A common pathogenic mechanism is further supported by the consistent involvement of NOX4 in mitochondrial dysfunction, which is induced by inflammatory cytokines or disruption of electron transport chain in astrocytes [52, 53] and, as we have shown in our study, by mitochondrial pore formation with BAX in neurons. Research into the complex interplay between neuronal and glial NOX4 in the context of PRL-mediated antioxidant neuroprotection is needed. Given our recent findings on PRL’s antioxidant properties in astrocytes [33], it would be interesting to investigate how this protective effect is related to the inhibition of NOX4-mediated mitochondrial impairment pathways.

Contrary to previous findings by Zhang et al. (2022) [54], our study did not find a significant role for NOX2 in neuronal ROS production and apoptotic cell death. Basal *Nox2* expression was very low in hippocampal neurons (Ct > 35) and was not significantly altered by H_2_O_2_ or PRL treatments (S2 Fig). Even though PRL may influence NOX2 activity, the absence of a high basal level of mRNA suggests a limited role for NOX2 in our experimental model. A possible explanation for the discrepancy between our findings and those of Zhang and colleagues could be the differences in neuronal culture type and developmental stage. While Zhang et al. used cortical DIV6 neurons, our study employed DIV10 hippocampal neurons. Previous research has indicated that NOX2 expression can decline in primary cultures of granular neurons as they mature [55]. Given our aim to study mature neurons with well-established connections, this might contribute to the low levels of NOX2 detected in our study. Future studies investigating the expression pattern of NOX2 in hippocampal primary cultures and the interplay between PRL, NOX2, and NOX4 *in vivo* could provide further mechanistic insights. Additionally, exploring the potential broader effects of PRL on NOX2 during neuroinflammation in neurons and microglia is warranted. NOX2 is a primary source of ROS in neurons associated with inflammatory conditions [56], and PRL can induce NOX2 expression and activity in isolated leukocytes [24, 25].

Overall, these findings establish a pivotal role for NF-κB in PRL’s neuroprotective mechanism and identify NOX4 as a novel downstream effector of NF-κB signaling in hippocampal neurons under oxidative stress conditions. Further exploration is needed to elucidate the precise mechanisms by which NF-κB downregulates NOX4 expression. One potential mechanism involves the JNK/c-Jun signaling pathway, since H_2_O_2_ induces the activation of this pathway in primary cortical neurons and c-Jun activation has been shown to promote NOX4 transcription in cultured endothelial cells [32, 57]. NF-κB, on the other hand, can inhibit JNK activation [58]. Investigating this pathway could provide valuable insights into the molecular mechanisms of PRL’s neuroprotective effect.

it is important to acknowledge that our study was conducted *in vitro*, and further *in vivo* research is necessary to fully understand the therapeutic potential of PRL in NOX inhibition in the hippocampus. This is particularly relevant considering that NOX activity has been repeatedly implicated in the progression of neurodegenerative conditions [52, 59, 60]. PRL can enter the cerebrospinal fluid by PRL receptor-mediated transport through the choroid plexus [61]. However, the rapid activation of hypothalamic neurons minutes after peripheral PRL administration, as evidenced by STAT5 phosphorylation [62], suggests that its primary route into brain neurons is likely the direct access from capillary blood via an unidentified PRL receptor-independent transporter across the blood-brain barrier [61, 62]. The exact mechanism by which PRL gains access to distant brain regions like the hippocampus remains unclear. It is uncertain whether PRL is released from capillaries into the extracellular space to diffuse until it reaches its cell target. Further investigations are needed to elucidate the precise mechanisms involved in PRL transport within the CNS.

Despite the limited data on hippocampal PRL signaling and ROS neurodegenerative diseases, the neuroprotective effects of peripheral PRL administration in the hippocampus of an Alzheimer’s disease model induced by intracerebroventricular injection of streptozotocin [63] support the hypothesis that PRL may play a protective role against oxidative stress in this brain region. Streptozotocin-induced Alzheimer’s disease models are known to increase brain oxidative stress [64], reinforcing the relevance of PRL’s antioxidant properties in this context. While translating our findings into therapeutic interventions remains a challenge, intranasal administration of PRL has shown promising results in neuroprotection in stroke models [65]. Future studies are warranted to evaluate the efficacy of this non-invasive route for delivering PRL in other neurodegenerative disease models and, ultimately, in humans.

Although elevated levels of PRL (hyperprolactinemia) can have adverse effects, such as decreased libido, amenorrhea, infertility, and breast-related issues [66], discrete increases in serum PRL display beneficial effects with reduced side effects [67]. In some cases, hyperprolactinemia associated with antipsychotic treatment has demonstrated antioxidant properties. This type of hyperprolactinemia has been linked to reduced oxidative stress markers (plasma MDA and H_2_O_2_), suggesting potential neuroprotective benefits for individuals with psychotic disorders [68]. Similar observations have been made in physiological hyperprolactinemia, such as lactation, where lower levels of lipid peroxidation in the hippocampus are observed in lactating rats [69]. While future research is necessary to find optimal conditions for PRL pharmacological treatment, these findings highlight the potential benefits of PRL in neuroprotection.

## Supporting information

S1 Fig BAY 11–7082 dose-dependent effect on neuronal viability. Treatment of hippocampal neurons with BAY 11–7082 at DIV10 induced a dose-dependent reduction in cell viability as measured by the MTT assay 24 h later. Statistical analysis revealed a significant reduction in cell viability at concentrations of 6 and 10 μM BAY 11–7082, while a concentration of 3 μM was determined to be non-toxic. *p<0.05, ****p<0.0001 vs vehicle.
(TIF)

S2 Fig Effect of PRL on the transcription of *Nox2* gene in mouse hippocampal neurons. Hippocampal neurons were pre-incubated for 24 h with 100 nM prolactin (PRL) or vehicle, followed or not by treatment with 3 μM of the nuclear factor kappa B (NF-κB) inhibitor BAY-117082 (BAY) 1 h before treatment with 100 μM H_2_O_2_ or vehicle for 24 h. Quantitative RT-PCR was used to measure mRNA levels of *Rbfox3* (NeuN) and *Nox2* in hippocampal neurons treated with vehicle or various experimental conditions. Data were normalized to *Hprt* housekeeping gene expression and further normalized to *Rbfox3* expression in control hippocampal neurons (n = 3–4).
(TIF)
