# Supplementary figures and images for: Prolactin protects hippocampal neurons against H2O2-induced neurotoxicity by suppressing BAX and NOX4 via the NF-κB signaling pathway
Source: PLoS One. 2024 Nov 5;19(11):e0313328. doi: 10.1371/journal.pone.0313328 (PMC11537405; doi:10.1371/journal.pone.0313328)

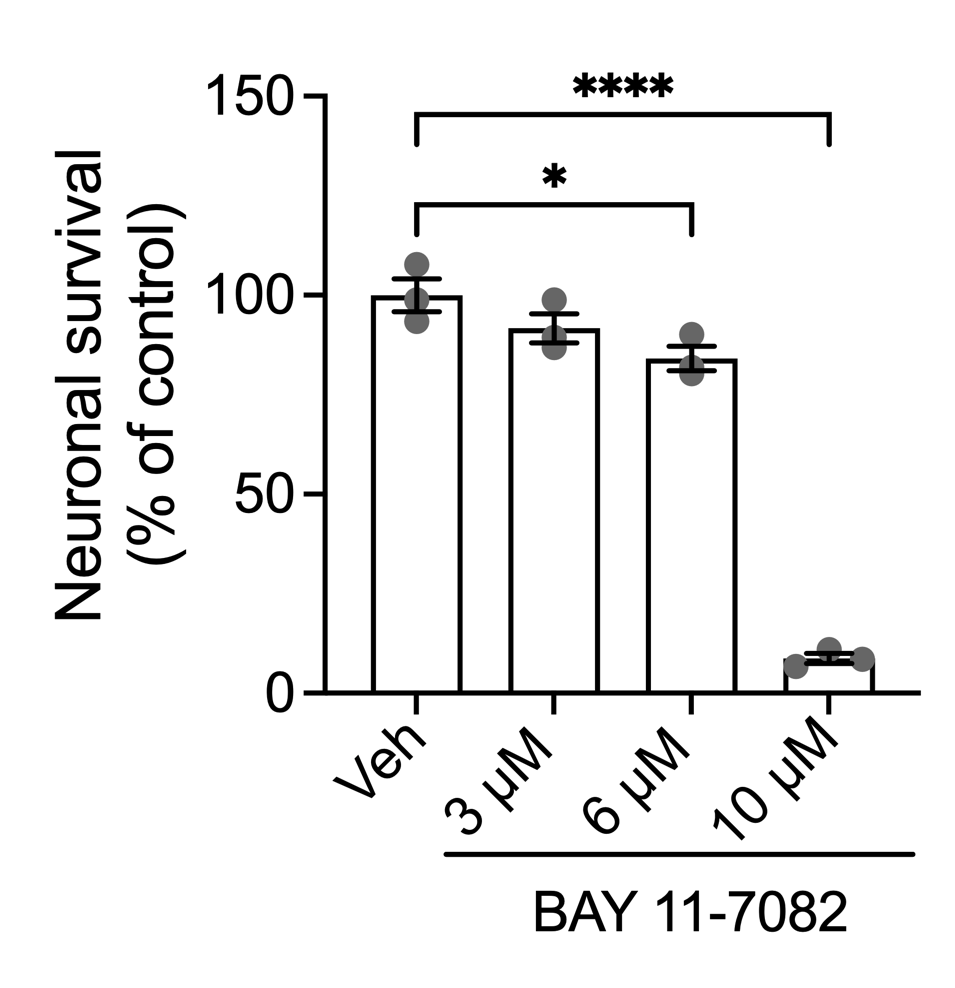

Supplement: S1 Fig — Treatment of hippocampal neurons with BAY 11–7082 at DIV10 induced a dose-dependent reduction in cell viability as measured by the MTT assay 24 h later. Statistical analysis revealed a significant reduction in cell viability at concentrations of 6 and 10 μM BAY 11–7082, while a concentration of 3 μM was determined to be non-toxic. *p<0.05, ****p<0.0001 vs vehicle. (TIF) [file pone.0313328.s001.tif]

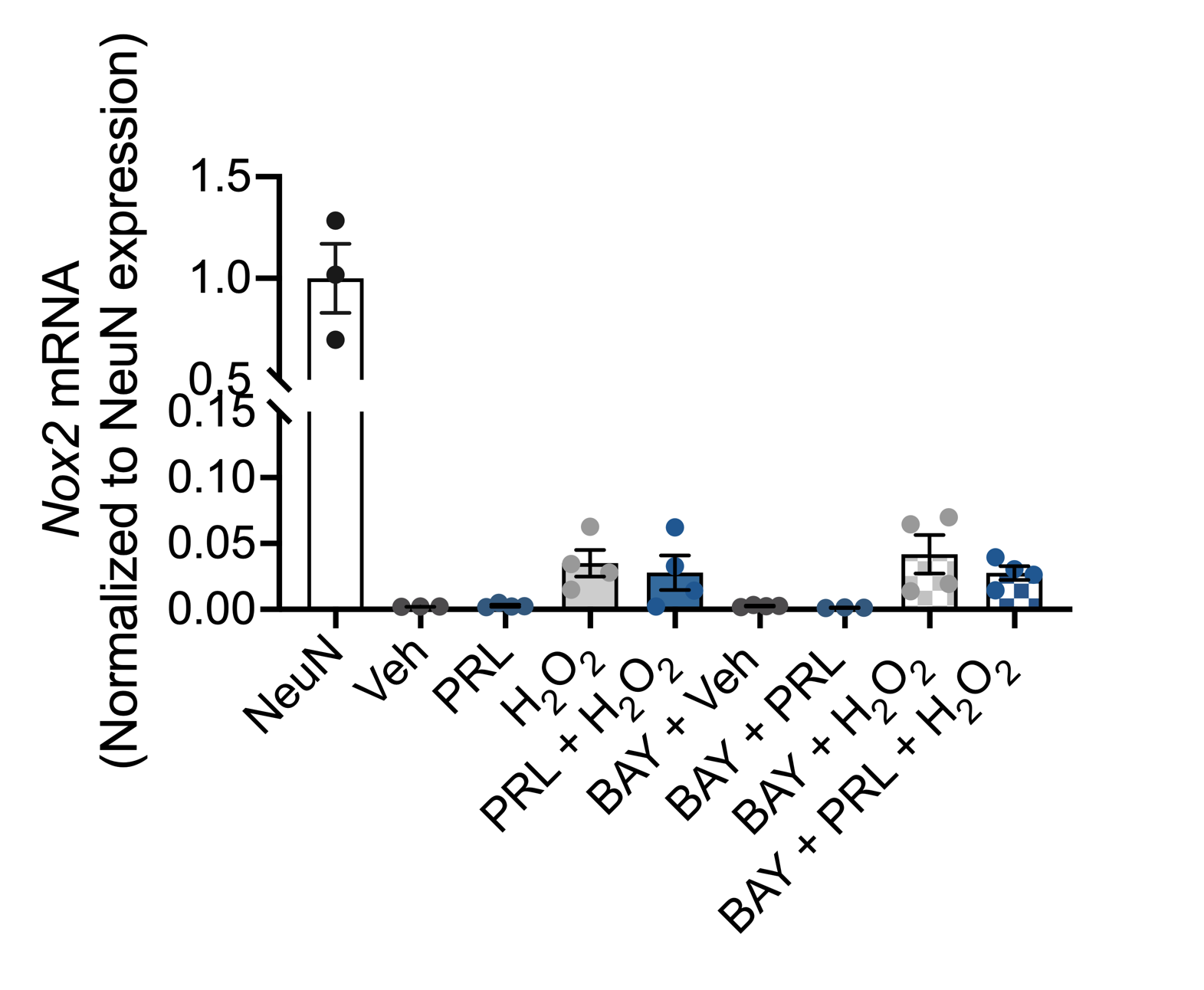

Supplement: S2 Fig — Hippocampal neurons were pre-incubated for 24 h with 100 nM prolactin (PRL) or vehicle, followed or not by treatment with 3 μM of the nuclear factor kappa B (NF-κB) inhibitor BAY-117082 (BAY) 1 h before treatment with 100 μM H2O2 or vehicle for 24 h. Quantitative RT-PCR was used to measure mRNA levels of Rbfox3 (NeuN) and Nox2 in hippocampal neurons treated with vehicle or various experimental conditions. Data were normalized to Hprt housekeeping gene expression and further normalized to Rbfox3 expression in control hippocampal neurons (n = 3–4). (TIF) [file pone.0313328.s002.tif]
